# Computer-Implemented Articulatory Models for Speech Production: A Review

**DOI:** 10.3389/frobt.2022.796739

**Published:** 2022-03-08

**Authors:** Bernd J. Kröger

**Affiliations:** Department of Phoniatrics, Pedaudiology, and Communication Disorders, RWTH Aachen University, Aachen, Germany

**Keywords:** articulatory model, speech production, biomechanical model, vocal tract, speech acoustics

## Abstract

Modeling speech production and speech articulation is still an evolving research topic. Some current core questions are: What is the underlying (neural) organization for controlling speech articulation? How to model speech articulators like lips and tongue and their movements in an efficient but also biologically realistic way? How to develop high-quality articulatory-acoustic models leading to high-quality articulatory speech synthesis? Thus, on the one hand computer-modeling will help us to unfold underlying biological as well as acoustic-articulatory concepts of speech production and on the other hand further modeling efforts will help us to reach the goal of high-quality articulatory-acoustic speech synthesis based on more detailed knowledge on vocal tract acoustics and speech articulation. Currently, articulatory models are not able to reach the quality level of corpus-based speech synthesis. Moreover, biomechanical and neuromuscular based approaches are complex and still not usable for sentence-level speech synthesis. This paper lists many computer-implemented articulatory models and provides criteria for dividing articulatory models in different categories. A recent major research question, i.e., how to control articulatory models in a neurobiologically adequate manner is discussed in detail. It can be concluded that there is a strong need to further developing articulatory-acoustic models in order to test quantitative neurobiologically based control concepts for speech articulation as well as to uncover the remaining details in human articulatory and acoustic signal generation. Furthermore, these efforts may help us to approach the goal of establishing high-quality articulatory-acoustic as well as neurobiologically grounded speech synthesis.

## Introduction

**FIGURE 1:**
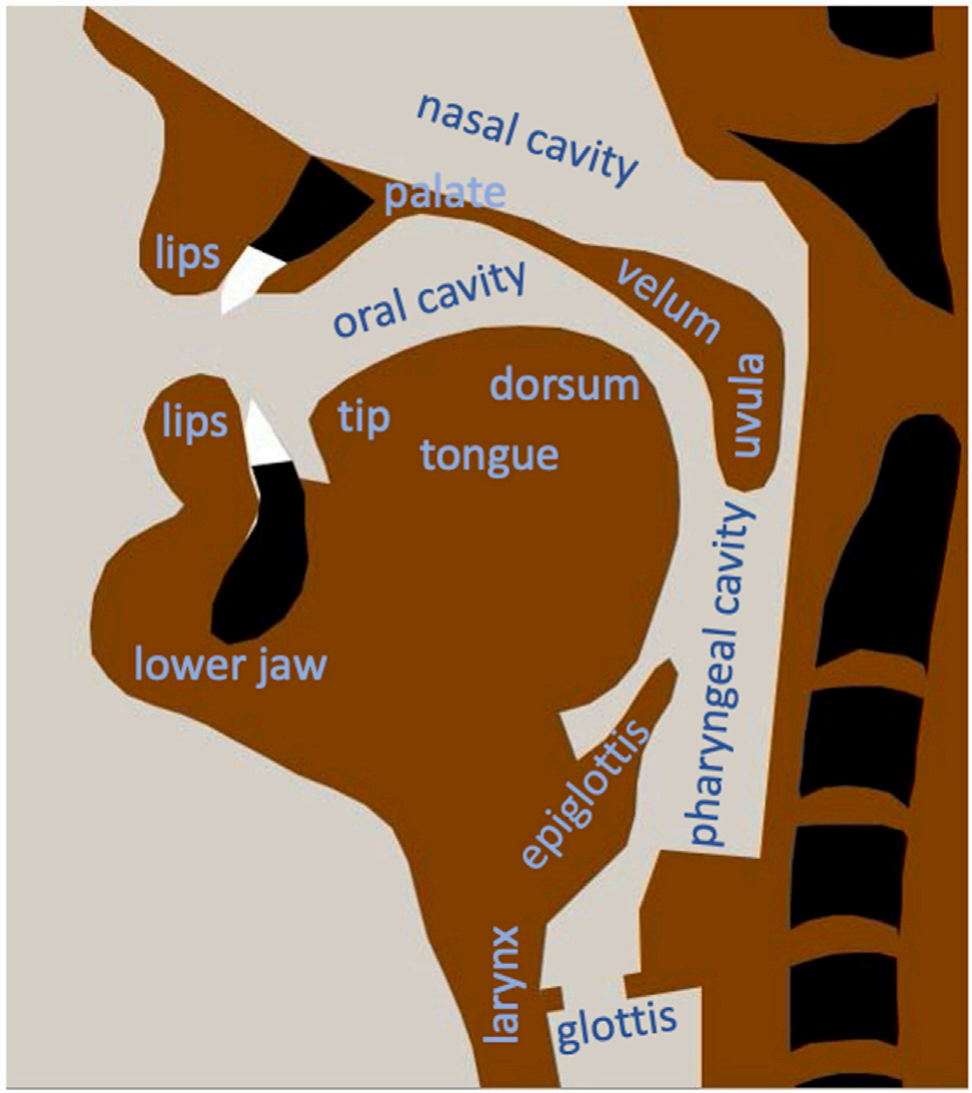
Midsagittal view generated using the two-dimensional articulatory model of Kröger et al. (2014).

An articulatory model is a quantitative computer-implemented emulation or mechanical replication of the human speech organs. It can be extended towards an articulatory-acoustic model if in addition an acoustic speech signal is produced based on the geometrical information provided by the articulatory model. Thus, the term articulatory model will include articulatory-acoustic models in this paper. The speech organs modeled in these approaches can be divided in sub-laryngeal, laryngeal, and supra-laryngeal organs. The sub-laryngeal system comprising lungs and trachea provides subglottal pressure and sufficient airflow for speaking, the laryngeal system provides the phonatory signal (primary source signal), and the supra-laryngeal system comprising pharyngeal, oral, and nasal cavities and comprising the articulators for modifying the shape of the pharyngeal and oral cavity, i.e., lower jaw, lips, and tongue, modify the phonatory signal and generates secondary source signals (frication noise) in case of the occurrence of appropriate constrictions. It is the main task of an articulatory model to produce natural articulatory speech movements based on articulatory control commands and in case of an articulatory-acoustic model in addition to generate an understandable and natural sounding acoustic speech signal. In case of the sub-laryngeal system the breathing activity during speech needs to be specified as a basis for calculating subglottal pressure and airflow. In case of the laryngeal system vocal cord tension and aperture needs to be specified as a basis for calculating the vocal fold vibrations and the phonatory acoustic signal. In case of the supra-laryngeal system a succession of vocal tract shapes (cavity shapes of oral, nasal, and pharyngeal cavity, Figure 1) needs to be specified for calculating the resulting acoustic speech signal.

Articulatory models mainly serve as a research tool 1) for defining a set of significant articulatory control parameters which is capable to control an articulatory model in all its aspects during speech production, 2) for verifying hypotheses concerning the neuromuscular and biomechanical properties of speech articulators, and 3) in case of articulatory-acoustic models to test acoustic and aerodynamic theories of speech production for generating realistic acoustic output based on the geometric data provided by the model.

First articulatory models were mechanical devices (e.g., Kempelen’s speaking machine; von Kempelen, 1791, see also Dudley and Tarnoczy, 1950). Because most computer-implemented articulatory speech synthesizers currently do not allow real-time signal generation and because of the advance in developing anthropomorphic systems during last decades, the existence of robotic-mechanical reconstruction approaches for the human vocal tract, allowing a direct aerodynamic and acoustic signal generation should be mentioned here as well (e.g., the WASEDA anthropomorphic talking robot; Fukui et al., 2009). But these approaches still have difficulties to mimic the neuromuscular and biomechanical details of the human vocal apparatus and thus to model the geometrical details of articulators, of vocal tract shapes, and of articulator movements.

In this paper we focus on computer-implemented models beginning with models developed in the 1960s/1970s up to contemporary articulatory models and synthesizers which are developed to approach the goal of producing high-quality articulatory-acoustic signals and/or to reproduce the neuromuscular and biomechanical properties of articulators as close as possible. We include articulatory models with and without an acoustic module because some approaches mainly concentrate on the neuromuscular and biomechanical aspects while other approaches concentrate on the generation of acoustically relevant vocal tract geometries. Moreover, many articulatory models focus exclusively on modeling the supra-laryngeal part of articulation, especially if an acoustic model part is not included. These articulatory models are included here as well.

## Features for Differentiating Computer-Implemented Articulatory Models

A (not necessarily complete) list of well published articulatory models is given in Table 1. These models can be differentiated with respect to a set of model features (columns of Table 1).

**TABLE 1:** List of computer-implemented articulatory models (rows) and criteria for differentiating articulatory models with respect to several features (rows; see text).

	Dim.	Biomechanical vs. geometrical vs. statistical	Number of control parameters	Data	Acoustic model	Complete VT	Dynamic vs. static, all sound or less, syllables	Major goal
Badin et al. (2002)	3D	statistical, linear component analysis	low (<10)	static MRI plus video facial data	no	yes	dynamic	identifying phonetic and biomechanical interpretable model control parameters
Beautemps et al. (2001)	2D	statistical, linear component analysis	low (9)	cine X-ray plus video facial data	yes	yes	dynamic	identifying the degrees of freedom (i.e., the number of control parameters) for an articulatory model (statistical model)
Birkholz and Jackel (2003), Birkholz (2013)	3D	geometric; parametric	middle (15)	static MRI data	yes	yes	dynamic	high quality speech synthesis
Buchaillard et al. (2009)	3D	muscle force model; biomechanical tissue model	middle (11)	static MRI and CT data for vowels (geometries); EMG data (muscle activation)	yes	yes	static V-sounds	muscle force levels for different French vowels
Coker (1976)	2D	geometric; parametric	low (<10)	static X-ray data	yes	yes	dynamic	speech synthesis by rule; developing an approach for articulatory commands
Dang and Honda (2004)	2D	muscle force model; biomechanical tissue model	middle (10 for tongue)	static MRI data	no	tongue + VT walls	movements towards V- and C-sound equilibrium positions	identifying agonist-antagonist muscle groups (muscle synergies) for V- can C-sounds
Engwall (2003)	3D	statistical; ordered linear factor analysis	low (6 for tongue)	static MRI plus EMA, EPG	no	tongue	static V- and C-sounds VC-sequences with C = fricative	identifying kinematic model control parameters; developing methods for including EMA and EPG data for modeling tongue movements
Harandi et al. (2015), Harandi et al. (2017)	3D	muscle activation + force model; biome-chanical tissue model	high (21 for tongue and jaw)	tagged MRI plus cine MRI data	formants	tongue + VT walls	tongue forward-backward movement	specifying speaker-specific muscle activation patterns based on tagged and cine MRI data
Henke (1966)	2D	geometric; parametric	Non-parametric “goal-seeking” approach	cine X-ray data	transfer function	yes	dynamic	specifying control concepts for articulatory movements and modeling coarticulation
Iskarous et al. (2003)	2D	geometric; parametric	low (<10)	static X-ray data, ultrasound, static MRI	yes	yes	dynamic	research tool; testing gesture patterns by perception
Kröger et al. (2014)	2D	geometric; parametric	low (<10)	static MRI data	yes	yes	dynamic	midsagittal views of dynamic articulation for teaching and as tool in speech therapy
Maeda (1979), Maeda (1990), Boë et al. (1995)	2D	statistical; principal component analysis; growth model	low (7), see also Toutios et al. (2011)	cine X-ray data	yes	yes	dynamic	research tool; identification of model control parameters
Mermelstein (1973), Rubin et al. (1981)	2D	geometric; parametric	low (<10)	static X-ray data	yes	yes	VCV-sequences	VCV-sequences; coarticulation; speech synthesis; research tool
Perrier et al. (2003)	2D	muscle activation + force model (lambda model); biomechanical tissue model	low (<10 for tongue)	qualitative comparison with CVC movement data extracted from literature	no	tongue	VCV-sequences	VCV-sequences; C = velar consonant; tongue body movement during C (loops)
Sanguineti et al. (1998)	2D	muscle activation + force model (lambda model); biomechanical tissue model	middle and low (17 muscles -> 6 factors explain a variance of 75%; tongue + jaw + hyoid)	cine X-ray data	no	tongue, hyoid, larynx, lower jaw	periodic jaw and tongue movements	organization of control signals; dynamic behavior of articulators; identifying muscle synergies
Serrurier et al. (2019)	2D	statistical; principal component analysis; speaker-specific	middle (14)	static MRI data	no	yes	static V- and C-sounds	estimation of control parameters; based on 11 different speakers; generating individual models and a mean speaker model
Serrurier and Badin (2008)	3D	statistical; generic surface triangular mesh; principal component analysis	low (2 for velum)	static MRI and CT plus EMA data	yes	velum + naso-pharyngeal wall	static V- and C-sounds	identifying geometric model control parameters; modeling velum movements using additional EMA data; resynthesis of nasals
Stark et al. (1999)	2D	geometric	low (<10)	cine X-ray data	yes	yes	dynamic	speaker-specific vocal tract geometries for short sound sequences
Stone et al. (2018)	1D	parametric area-function	middle (16)	static MRI data	yes	yes	dynamic	high quality and real time speech synthesis
Story and Titze (1998), Story (2005), Story et al. (2018)	1D	parametric area-function model; speaker-dependent; growth	low (<10) for static vowel model; middle (14) for dynamic model	static CT and MRI data	yes	area functions including nasal tract	dynamic (VV, VCV and VCCV utterances) or static V- and C-sounds	articulatory-acoustic relations for males/females for newborns/children/adults; high-quality speech synthesis of isolated sounds and of sound sequences
Wilhelms-Tricarico (1996)	3D	muscle activation + force model; biomechanical tissue model	low (<10 at higher control level); middle (<20 at lower control level; tongue)	static MRI data	no	tongue	static tongue configurations	physiologically based computer simulation of speech production; research tool

Two-dimensional models (e.g., Mermelstein, 1973; Maeda, 1990; Stark et al., 1999; Beautemps et al., 2001; Iskarous et al., 2003) generate mid-sagittal shapes of the vocal tract. While this geometrical information is the most relevant 2D-information for many speech sounds there exist sounds like the laterals for which the midsagittal information is misleading. Laterals exhibit a midsagittal closure while the lateral parts of the tongue produce an opening for laminar air flow. Traditionally the area function is used as relevant information for calculating vocal tract acoustics from geometrical data. The area function represents the succession of cross-sectional areas occurring between glottis and mouth, perpendicular to the airflow within the vocal tract (area in cm^2^ as function of distance from glottis). There exist approaches to approximate area functions from midsagittal distances (Heinz and Stevens 1964; Perrier et al., 1992) but these approaches only approximate area functions roughly because the shape of the cross-sectional area of the vocal tract cavity perpendicular to the airflow varies strongly between glottis and mouth. Three-dimensional models (e.g., Buchaillard et al., 2009; Harandi et al., 2017) provide the full spatial information and are capable to calculate correct area functions in a straightforward way. But the goal of most current 3D-models is not the generation of acoustic speech signals but the detailed modeling of neuromuscular and biomechanical details of articulators. Moreover 3D-models are able to incorporate sagittal asymmetries in articulation which occur to a certain degree in normal as well as in disordered speech production. Beside 2D-models representing the midsagittal plane and beside 3D-models representing the complete shape of the vocal tract, even 1D-models are available, which directly calculate and process the acoustically relevant area function (Story and Titze, 1998; Story, 2005; Stone et al., 2018; Story et al., 2018). “One dimension” in this case means that these models do not calculate the articulator shapes in a two-dimensional midsagittal plane or in the three-dimensional space, but just calculate one parameter, i.e., the (one-dimensional) distance between articulator and vocal tract wall as function of distance from the glottis along the midline of the vocal tract from glottis to mouth (e.g., Stone et al., 2018, p. 1381). These models are focusing directly on the acoustic effects of speech articulation.

First computer-implemented articulatory models were geometrical models (Henke 1966; Mermelstein 1973; Coker 1976; Rubin et al., 1981). Geometrical models are defined by making a priori assumptions concerning the basic geometries (parts of circles, straight lines) for constructing articulator shapes and concerning the set of control parameters. One of the most detailed geometrical 2D-model is that published by Iskarous et al. (2003). A further very detailed geometrical 3D-model has been developed by Birkholz and Jackel (2003). Both models are able to produce fluent speech and can be adapted to vocal tract geometries of different speakers. One main goal of geometrical models is to deliver a simple but phonetically and/or linguistically meaningful set of control parameters. Thus, the model of Iskarous et al. (2003) directly parameterizes degree and location of consonantal constrictions and the high-low, front-back, and rounded-unrounded dimension for vowels. Control parameter estimation can be done for this kind of model by fitting model vocal tract shapes to imaging data (contours stemming from X-ray, CT, EMA, or MRI measurements of natural speakers, see: Toutios et al., 2011; Ramanarayanan et al., 2013; Narayanan et al., 2014).

Statistical models are directly based on defined sets of imaging data. No a priori assumptions are made concerning articulators and their control parameters. Control parameters are defined here by using statistical procedures evaluating the variance of vocal tract shapes occurring in the data set. The data set can be a set of continuous movement data of whole sentences or a set of static imaging data representing a list of sound targets. The location of predefined flesh or tissue points of the articulators and vocal tract walls is extracted for each image and a principal component analysis is done on these sets of flesh point locations (Maeda, 1990; Badin et al., 2002; Engwall, 2003; Serrurier et al., 2019). In case of the Maeda model 88% of the variance of the flesh points, derived from a data set of 10 sentences uttered by a French speaker (519 images in total), can be explained by seven articulatory parameters, which are directly interpretable as physiological parameters for lips, tongue, jaw, and larynx (Boë et al., 1995).

The goal of geometric and statistical models is to parameterize vocal tract shapes with as few parameters as possible (see column 4 of Table 1: number of control parameters <10 for most of these models), but with enough flexibility to fit different speakers (adaptivity) as well as to fit the whole variety of vocal tract shapes occurring for each speaker in fluent speech. Most of the current statistical models are 2D-models (see Tab. 1) but a few 3D-models exist as well (Badin et al., 2002; Serrurier and Badin, 2008).

Biomechanical models approximate the anatomy and biomechanical properties of all vocal tract articulators. Its time-variant input is a vector of muscular activation levels leading to speech articulator movements and producing a succession of articulator displacements and thus of vocal tract shapes over time. Here, the body of articulators like tongue, lips, or velum is subdivided into 2D- or 3D-finite elements with simple shapes. These elements are defined by a mesh of corner points. The current location or displacement of these tissue or mesh points and its displacement velocity and acceleration specifies all articulator movements. The forces acting on each mesh point and leading to mesh point movements result from active forces generated by the muscles and from passive forces resulting from biomechanical soft tissue properties. Some biomechanical models are 2D-models (e.g., Sanguineti et al., 1998; Perrier et al., 2003; Dang and Honda, 2004) but most biomechanical models are 3D-tongue models (e.g, Wilhelms-Tricarico, 1996; Buchaillard et al., 2009) or complete 3D-vocal tract models (e.g., Harandi et al., 2017). The definition of the 2D- or 3D-finite element mesh is typically based on static X-ray or MRI data displaying the neutral or rest positioning of articulators. Because the set of muscles and its function during speech is complex, muscles are combined to synergetic acting muscle groups in many biomechanical models. These combinations of muscles to muscle groups allows a direct control of speech-like articulator movements like vocalic up-down and forward-backward movements of the tongue dorsum or like consonantal tongue tip elevation (e.g., Dang and Honda, 2004). The number of control parameters in general is higher in case of biomechanical models (see column 4 of Table 1: number of control parameters is in the range “middle” or “high”, i.e., >10 control parameters), in order to be able to include all muscle activity values of all relevant muscles or muscle groups controlling different articulators.

The data set on which a model is based is of great importance for its quality and its empirical realism. In case of most models, static magnetic resonance imaging (MRI) data, static 2D X-ray, or static 3D-computer tomography (CT) data are used which display target vocal tract shapes of vocalic and consonantal speech sounds of one or more speakers (e.g., Mermelstein, 1973; Beautemps et al., 2001; Birkholz and Kröger, 2006). Dynamic X-ray movement data (cine-radio data; cineradiography), i.e., a succession of vocal tract shapes for complete utterances are rare because of ethical reasons due to the high radiation exposure. MRI movement data (cine MRI)—which can be gathered with less health risks–are hard to acquire with a sufficient spatio-temporal resolution but are currently used more and more (Harandi et al., 2017; Carignan et al., 2021). In order to check movement features of a model EMA data (electromagnetic articulography), EMA data in combination with EPG data (electropalatography), or X-ray microbeam data can be used but these data give only selective punctual spatial information concerning the vocal tract shape (Westbury et al., 1990; Wrench and Hardcastle, 2000; Toutios et al., 2011). Moreover, tagged MRI in combination with cine MRI (2D) is capable of tracking movements of tissue points on the surface as well as inside an articulator and thus can give information concerning the displacement of finite element mesh points during connected speech (Stone et al., 2001; Harandi et al., 2017).

Some articulatory modeling approaches try to approach the goal not just to generate vocal tract shapes but in addition to generate acoustic speech signals based on the input control information (e.g., Rubin et al., 1981; Maeda, 1990; Iskarous et al., 2003; Birkholz, 2013; Story et al., 2018). The goal of older models was to reach high-quality speech synthesis using simple articulation-based control concepts. Later models mainly aim for unfolding articulatory-acoustic relations or want to check whether the implemented control concept results in correct audible speech movements. Acoustic models implemented in 2D-articulatory models calculate mainly pressure and flow changes along the direction of airflow and can be called one-dimensional acoustic models while 3D-articulatory models can incorporate more complex 2D- or 3D-acoustic models as well (Mullen et al., 2007; Speed et al., 2013).

Most articulatory models, especially those which aim to generate vocal tract shapes as well as acoustic speech signals model all articulators and thus generate the complete vocal tract shape. These models are mainly geometrical or statistical models (e.g., Rubin et al., 1981; Maeda, 1990; Iskarous et al., 2003; Birkholz, 2013) while most of the biomechanical models concentrate on modeling the tongue, its movements and its neuromuscular structure (e.g., Wilhelms-Tricarico 1996; Engwall, 2003; Perrier et al., 2003; Dang and Honda, 2004). Some models do not calculate an audible acoustic signal but the acoustic transfer function of the vocal tract or the formant pattern (Henke, 1966; Harandi et al., 2017).

A further criterion for differentiating articulatory models is whether they are able to generate static vocal tract shapes only or whether they are able to generate articulatory movement patterns. Most of the articulatory models generate dynamic vocal tract articulator movements. But some models just generate static vocalic vocal tract shapes because they concentrate on research questions concerning vowel systems (e.g., Buchaillard et al., 2009) or they concentrate on the generation of static vowel and consonant vocal tract shapes because of research questions concerning language-specific sound systems (e.g., Serrurier et al., 2019).

**FIGURE 2:**
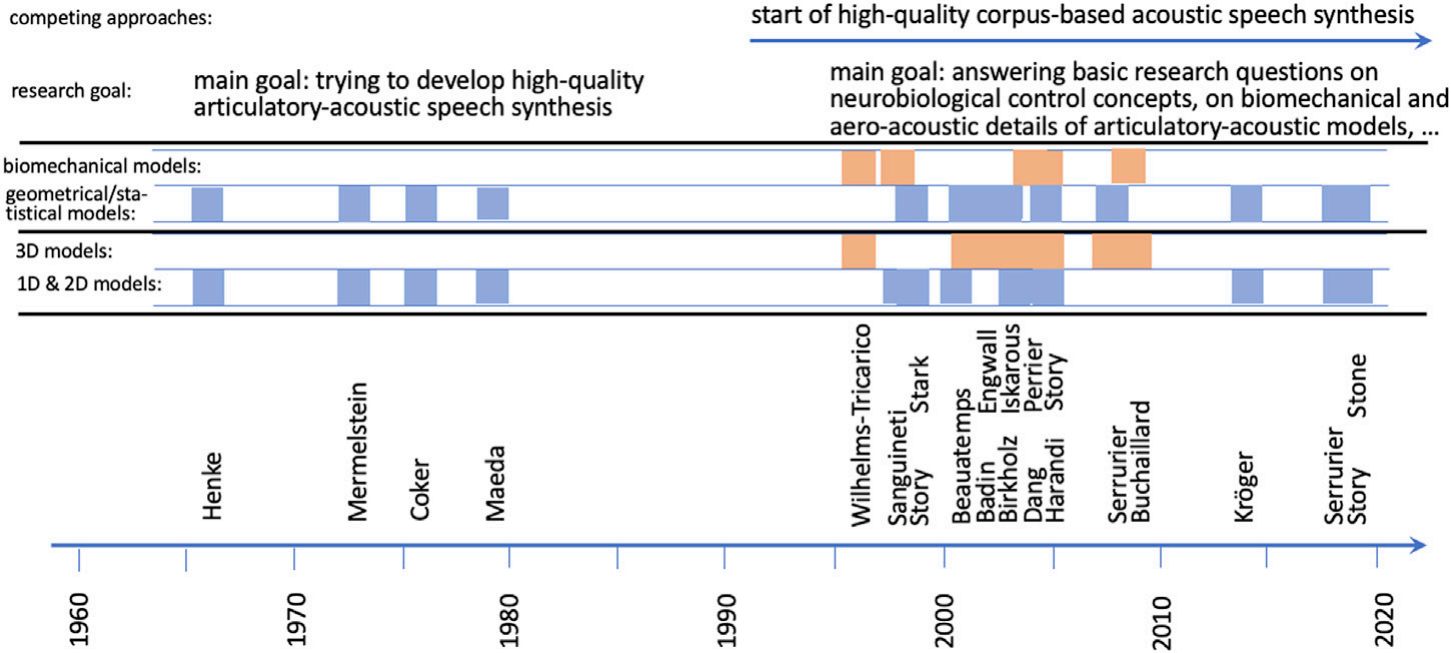
Visualization of the evolution of articulatory models over time. Models are cited here by the first author, as listed in Table 1. Main research goals and two criteria for differentiating articulatory models (biomechanical vs. geometrical and statistical models; 1D and 2D models vs. 3D models) are labeled and visualized.

The last criterion for differentiating articulatory models listed in Tab. 1 is the question concerning the primary research goal for which a model is intended to be used. As mentioned earlier one of the goals in the early years of computational articulatory models (earlier than the last decade of the 20th century, Figure 2) was to generate high-quality speech synthesis on the basis of a relatively restricted but phonetically linguistically relevant set of articulatory control parameters following a rule system of intuitive and linguistically motivated commands.

But this goal is not reached yet and the paradigm for developing articulatory models changed towards modeling speech articulators in more detail on the biomechanical level and to extract neurobiologically relevant higher-level control commands for the generation of the complex muscle activation pattern for all vocal tract articulators and thus for the generation of speech articulator movements. This branch of models can be labeled as neurobiologically and biomechanically realistic models.

One further research goal which can be focused on by using articulatory models including a neurobiologically realistic control concept is to model speech acquisition. Because it is not possible to uncover the exact neural learning processes during learning tasks like babbling (i.e., exploring all articulatory and acoustic capabilities of the own vocal tract) or during learning tasks like imitation (i.e., trying to learn words from a caretaker in order to be able to start first trials of speech communication) as well as to uncover the microscopic aspects of the development of the human nervous system during the first years of life where speech acquisition mainly takes place, neurobiologically inspired computational control models including articulatory models as front-end devices for the generation of articulatory movement patterns and acoustic speech-like (and later speech) signals are helpful. These models are able to mimic learning processes during speech acquisition including sensorimotor learning and thus to unfold potential neural processes of speech learning. A review of this research is given by Pagliarini et al. (2021). The articulatory models used in this research area are mainly the Maeda (1990) model which can be downloaded as VTCalcs software (see VTCalcs, 2021) and the Birkholz (2013) model which can be downloaded as Vocal Tract Lab (see VocalTractLab, 2021).

## Modeling Vocal Tract Aerodynamics and Acoustics: The Sound Sources

The quality of a synthetic acoustic speech signal strongly depends on the quality of the source signals. Sound sources occurring in speech production are the vibrating vocal folds leading to phonation in case of voiced sounds and further vocal tract constrictions leading to an increase in velocity of air flow and causing turbulent noise. While the vibrating vocal folds are labeled as primary sound source further constrictions within the vocal tract capable of producing turbulent noise are called secondary sound sources. Secondary sound sources appear e.g., in fricative sounds like/f/,/v/,/s/,/z/or/S/or/Z/(SAMPA symbols are used here, see SAMPA, 2021). Here, a vocal tract constriction is produced by an articulator approaching a vocal tract wall or a second articulator (e.g., lower lips approaching upper teeth or tongue tip approaching the alveolar ridge or hard palate) which causes an accelerated flow within and in front of the constriction (jet flow) and which leads to turbulent noise in front of the constriction. The glottis (glottal constriction) itself can produce turbulent noise as a side product during phonation or as a “stand alone sound” (glottal fricative/h/) if the vocal folds are adducted. Within the phonation mode the vocal folds exhibit oscillations leading to a (quasi-) periodic modulation of the glottal flow and subsequently to a (quasi-) periodic acoustic speech signal with a fundamental frequency reflecting the glottal vibration period. In order to model both types of sound sources adequately a detailed aerodynamic and aeroacoustic model needs to be incorporated as part of an articulatory speech synthesizer.

Aeroacoustics deals with the interaction of the flow mode of air (aerodynamics) and the sound mode of air (acoustics). While in case of aerodynamics an air volume is seen as incompressible including translational as well as rotational motion and where energy is transported by moving the whole air volume (convection), in case of acoustics an air volume is seen as compressible (local pressure and flow variations) and energy is transported here by sound wave propagation (e.g., Krane, 2005). While airflow (aerodynamics) is mainly considered as steady or quasi-steady (temporal variation of vocal tract constrictions like building, holding and release of a constriction appears in intervals of about 50 msec or longer and thus reflect a periodicity below 20 Hz), acoustic pressure and flow variations can be considered likewise as fast (above 20 Hz), but the transition from aerodynamics to acoustics in terms of frequency or periodicity is fluent. In case of primary and secondary sound sources especially the energy transfer from air flow to acoustics in case of secondary sound sources and from air flow to the mechanical system of the vocal folds in case of the primary sound source needs to be modeled in-depth.

Because current simulation models of sound sources especially in case of the generation of turbulent noise do not yet provide satisfactory auditory results a lot of research is done using mechanical aeroacoustic models and theoretical approaches for the primary and secondary sound sources (e.g., McGowan, 1988; Pelorson et al., 1997; Krane, 2005; McGowan and Howe, 2012; McPhail et al., 2019; Motie-Shirazi et al., 2021). New simulation approaches have been developed including more detailed knowledge concerning aeroacoustic phenomena concerning secondary sound sources (e.g., Howe and McGowan, 2005) as well as concerning the primary sound source (e.g., Schickhofer and Mihaescu, 2020; Schoder et al., 2020) but these approaches are computationally complex and not integrated in articulatory speech synthesizers so far. A well elaborated acoustic model for articulatory speech synthesis including all relevant loss mechanisms important for a correct modeling of vocal tract acoustics and for steady state aerodynamics and which can be implemented in a time-domain reflection-type line analogue without high computational costs has been developed by Liljencrants (1985) and Liljencrants (1989) and an equivalent frequency domain approach has been published by Sondhi and Schroeter (1987). Both approaches are still widely used in articulatory speech synthesis systems. These types of models produce acceptable acoustic signal quality in case of voiced sounds and they allow the integration of noise source generators for modeling fricative sounds, plosive noise bursts, and glottal noise. The noise source amplitude is controlled here by the Reynolds number and the spectrum of the noise source is white or colored noise depending on the place of articulation. While these systems are one dimensional (only calculating pressure and flow along the vocal tract cavity midline from glottis to mouth eventually branched for including the nasal cavity) current solutions based on fluid dynamic approaches which model the three-dimensional wave propagation within the vocal tract are available as well (e.g., Huang et al., 2002; Levinson et al., 2012). To this day only few acoustic models including a detailed aeroacoustic concept are designed for articulatory speech synthesis because of their computational effort (Pelorson et al., 1994; Sinder et al., 1998; Narayanan and Alwan, 2000; Birkholz et al., 2007; Zappi et al., 2016; Pont et al., 2018).

In case of the primary sound source, we can separate models directly prescribing a glottal cross-sectional area as function of time and models including a self-oscillating glottis module. While in the first category of models the glottal flow can be calculated directly (e.g., Titze, 1989; McGowan and Howe, 2012), a complete mechanical model of the vocal folds needs to be implemented in case of the second category of models in order to calculate the vocal fold vibration pattern and the glottal area as function of time first before the glottal air flow can be calculated. Here the oscillatory motion pattern of the vocal folds results from the interactions of this mechanical system with the aerodynamic system (self-oscillating glottis models, e.g., Ishizaka and Flanagan, 1972; Story and Titze, 1995; Avanzini et al., 2006; Tao et al., 2006; Elie and Laprie, 2016; Maurerlehner et al., 2021). In case of both models aeroacoustic effects are important mainly for shaping the waveform of the glottal flow (e.g., McGowan and Howe, 2012) but aerodynamic and aeroacoustic effects can influence the pressure distribution along the glottal constriction as well and thus influence the aerodynamic forces acting on the vocal folds and thus influencing the glottal area oscillation pattern (Pelorson et al., 1994; McPhail et al., 2019).

In order to be able to calculate the air flow in the vocal tract, first of all the subglottal pressure must be determined. The mean subglottal pressure occurring in speech production for example occurring over syllable-level time intervals is mainly a function of volume change of the lung during expiration (e.g., Tanihara et al., 2018) and together with the quasi-steady air flow it in addition depends on the mean aerodynamic resistance resulting from the glottal constriction and the vocal tract constrictions occurring downstream towards the mouth. Thus, a sub-laryngeal system including a lung model controlled by articulatory parameters like lung volume or lung force (McGowan and Saltzman, 1995) together with a laryngeal system including a physiologically based vocal fold model controlled by articulatory parameters like glottal aperture and vocal fold tension (e.g., Ishizaka and Flanagan, 1972) together with a supra-laryngeal articulatory-acoustic system including aeroacoustics as discussed above is needed in order to complete an articulatory-acoustic speech production model from the aerodynamic point of view.

## An Open Question: Muscle Groups, Muscle Activation Patterns, and Concepts for Articulatory Control Commands

**FIGURE 3:**
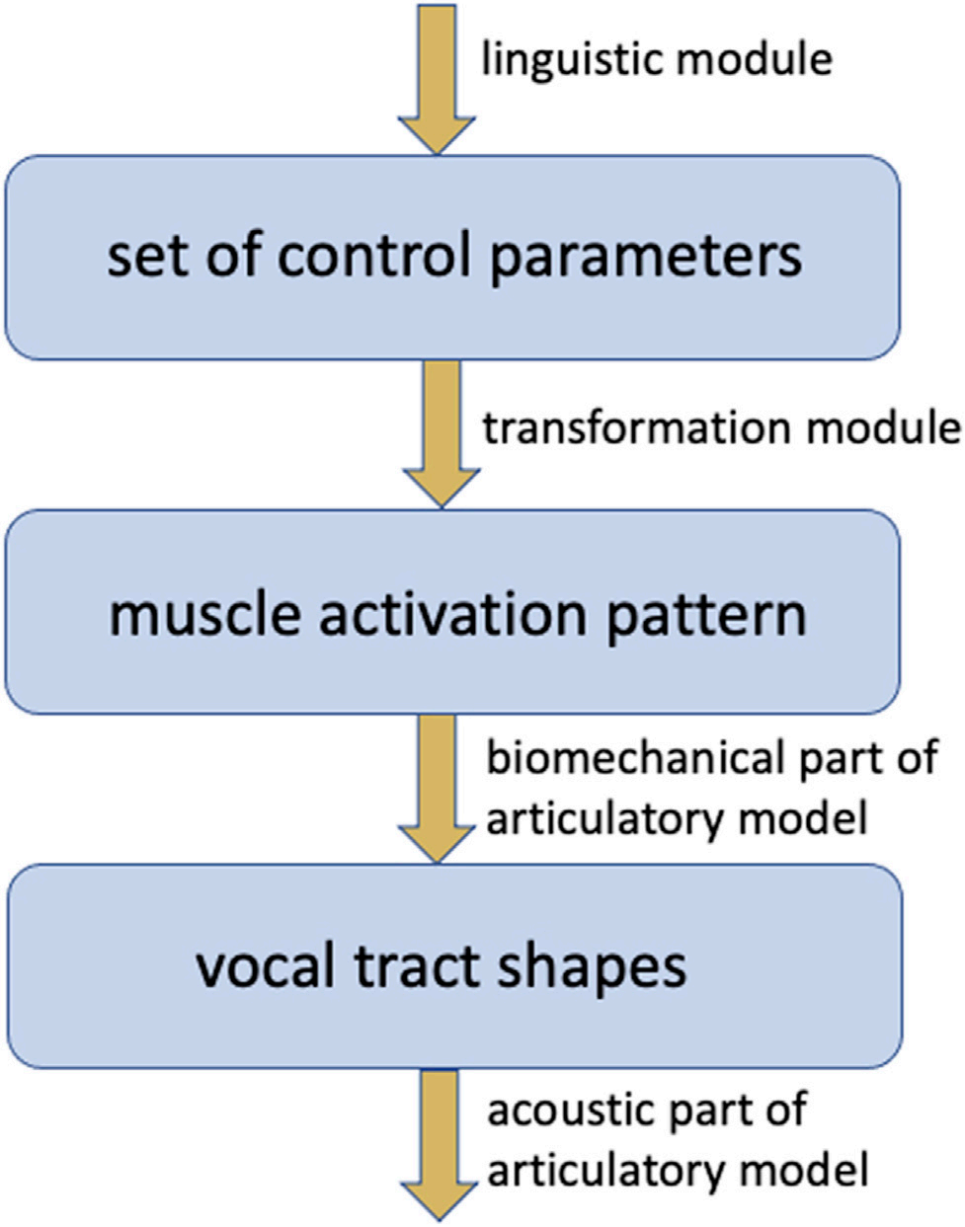
Hierarchical organization of articulatory models and their control modules and their levels of control (blue boxes).

A crucial problem concerns the set of parameters controlling an articulatory model. The set of control parameters should be as small as possible and should be intuitive in the sense that each parameter is interpretable at the articulatory-phonetic level (e.g., location and degree of consonantal constriction, vocalic dimensions like front-back, low-high, rounded unrounded). Moreover, this set of control parameters should be neurobiologically plausible. It should be able to extract this set of parameters from muscle activation patterns and vice versa, it should be possible to transform each pattern of control parameters into a muscle activation pattern which controls the articulators and thus completely describes the displacements or movements of all articulators (Figure 3). In a neurobiologically grounded modeling approach the activation of a higher level description of speech articulation (which should be transformable into a vector of model control parameter values for each point in time) occurs within the cortical premotor area while the more complex muscle activation pattern is activated within the primary motor area for each utterance.

It is not possible to extract such a set of neurobiologically plausible control parameter patterns in a straightforward way *in vivo* because we cannot uncover all details of the human cortical and subcortical neural network which is responsible for speech processing. A prerequisite for extracting control parameters indirectly from models is that we have available a detailed biomechanical articulatory model including a detailed modelling concept for articulator tissues and for all muscles controlling the articulators. If we can develop a control concept for such a model which leads to natural articulatory speech movement patterns and which exhibits different control parameters for different linguistic goals (e.g., consonantal versus vocalic movement patterns) it can be concluded that the appropriate set of control parameters is a promising candidate to be incorporated in a neural control concept.

A detailed model for relating muscle activation, muscle force, and muscle length is the λ model (Feldman et al., 1990) which has been applied to speech articulation (Sanguineti et al., 1998; Perrier et al., 2003). In this approach muscle activation results from an external activation control signal as well as from current muscle length and rate change of length. Thus, neuromuscular activation depends on a control signal (muscle activation patterns generated at the primary motor cortex) and on proprioceptive feedback. On this basis muscle force can be calculated from the external muscle activation control signal in combination with information about muscle length and its rate change. It is concluded that each muscle can be controlled effectively by specifying its threshold muscle length. This set of threshold muscle length, labeled as set of λ’s, determines the current equilibrium position of all articulators and is used in this approach as an equivalent to muscle activation patterns.

In all muscle-based biomechanical approaches (e.g., Wilhelms-Tricarico, 1996) including the λ model, active stress of muscle tissue results from neuromuscular activation. This stress is muscle force per cross-sectional area of all muscle fibers of a muscle and leads to muscle contraction, i.e., change in muscle length. Contrariwise, passive stress and strain of muscle tissue and other tissues inside and at the surface of articulators results from deformation.

It is assumed that the set of central control parameters is presumably organized into a number of different combinations of threshold muscle lengths and their change. Thus, different muscles or parts of muscles (macro-fibers of muscles) together form synergies for initiating movement primitives or elementary motor behaviors. Movement primitives are identified by Sanguineti et al. (1998) for different articulators like jaw and tongue dorsum. Perrier et al. (2003, p. 1589) defines the central control commands directly as sets of λ’s or as “targets” or “postures” which represent specific equilibrium positions for all articulators for different speech sounds. Movements are produced by continuously varying the set of λ’s from one sound target to the next sound target. Because the set of λ’s defines the location of all articulators completely, different sound targets need to be specified for consonants in order to reflect the coarticulatory influence of neighboring vowels while each vowel can be represented by one target. A concrete set of λ’s is estimated for three French vowel realizations of/i/,/a/and/u/for the tongue by Buchaillard et al. (2009). λ values are given here as a percentage of muscle length with respect to the muscles rest length defining the rest position of the tongue.

A set of movement primitives resulting from specific subsets of muscles or parts of muscles are called muscle groups is suggested by Dang and Honda (2004). These muscle groups are activated by defining co-contraction activation levels for agonist-antagonist pairs or triplets of muscles. Muscle groups and corresponding movement patterns are identified for independent tongue dorsum and tongue tip movements here (vocalic and consonantal movement primitives). This approach allows to specify subsets of muscles for example for defining vocalic tongue dorsum or consonantal tongue tip movements and consonantal movements can be superimposed on underlying vocalic movements in this approach.

Harandi et al. (2017) present a data driven approach for estimating muscle force and muscle activation patterns based on cine MRI and tagged MRI data. Here muscle activation patterns are estimated for the whole set of muscles for each point in time. Higher-level control concepts are not postulated.

A non-muscle-based model for controlling a geometrical articulatory model (Iskarous et al., 2003) is given by Ramanarayanan et al. (2013). Here, a pattern of linguistically defined gestures as postulated in the framework of Articulatory Phonology (Browman and Goldstein 1992) is activated for controlling all speech articulators. Here, gesture-based activation patterns establish the central control structure. But a (lower-level) biomechanical muscle-based activation level is not included. The approach directly defines (higher-level) activation patterns for gestures, which are later labeled as articulatory movement primitives by Ramanarayanan et al. (2013, p. 1378): “Articulatory movement primitives may be defined as a dictionary or template set of articulatory movement patterns in space and time, weighted combinations of the elements of which can be used to represent the complete set of coordinated spatio-temporal movements of vocal tract articulators required for speech production.” These movement primitives or gestures are defined as coordinative structures based on synergy principles by controlling the movements of one or more articulators and can be converted into movement pattern by using the task-dynamics approach (Saltzman and Munhall 1989). Based on an analysis of EMA and dynamic MRI data using matrix factorization techniques seven articulatory primitives are identified by Ramanarayanan et al. (2013), i.e., labial, apical and dorsal constriction gestures in context of front or back vowels. All models including detailed biomechanical muscle models and a muscular activation level are labeled in column 3 of table 1 as “biomechanical”.

A blueprint for the organization of neural control in speech production is given by the DIVA model (Directions Into Velocities of Articulators, see Guenther 2006, Bohland et al., 2010, and Guenther and Vladusich 2012). This approach differentiates a higher level neural representation of speech units like syllable called speech sound map located in the premotor cortex and a lower level neural representation of these units called articulatory map located in the primary motor cortex. Bohland et al. (2010) extended the DIVA approach towards the GODIVA-model (Gradient Order DIVA model) which in addition gives a detailed description of the planning process of articulation, i.e., how planning units like syllables, words or short phrases can be parsed from the flow of linguistic-phonological information. But the concrete implementation of the DIVA model leads to control signals for a statistical model (Maeda model, see Maeda, 1979, Maeda, 1990 and Boë et al., 1995) which does not control a detailed muscle-based biomechanical articulatory model.

## Discussion: Limitations and Future Directions for Modeling Speech Articulation

One of the main problems in developing articulatory models is the lack in articulatory data exhibiting a sufficient spatio-temporal resolution. Static MRI and X-ray (or CT) data give a sufficient spatial resolution in order to extract the surface shape of all articulators and vocal tract walls with a mean error of around 1 mm but a sufficient temporal resolution is only reached by some highly specialized research groups (e.g., Fu et al., 2017; Carignan et al., 2021). In case of midsagittal 2D-data a resolution of about 50 Hz (time interval of 20 msec) as is reported for cine X-ray tracking is not sufficient in case of tracking and modeling for example consonantal closing-opening movements. The temporal resolution for tracking speech movements should reach 10–5 msec (100–200 Hz). In the case of 3D-data most measurements extract static vocal tract geometries because the data acquisition procedure still takes seconds in case of MRI. In some laboratories cine 2D-MRI data can be acquired already with rates of 12.5–23 Hz (80–43 msec) with a spatial resolution of about 2.5 mm (Narayanan et al., 2014). Thus, EMA data are added in order to get a sufficient temporal resolution. Here, 12 flesh points or more can be tracked with a temporal resolution of 200 Hz and higher and with a spatial resolution below 1 mm (Richmond et al., 2011). EMA in principle is not limited to measuring 12 flesh points but gluing coils on articulators (tongue, lips) is tricky and limits the total number of coils that can be glued on. Moreover, EMA receiver coils with its diameter of about 3 mm may influence articulation. Recently even cine MRI increases dramatically in temporal resolution up to 50 Hz (20 msec) with a spatial resolution of 1.4 mm including a post-processing resampling procedure leading to an even higher temporal resolution up to a factor 10 (Carignan et al., 2021) or up to 166 Hz (6 msec) with a spatial resolution of 2.2 mm (Fu et al., 2017).

Current biomechanical models mainly concentrate on modeling jaw and tongue. A complete model including all articulators should be established, but the main problem with biomechanical models is its control. A huge number of muscles needs to be modelled and thus the muscle activation pattern controlling articulation over time is complex. It is assumed that this lower-level control information is generated from a higher-level motor command level, but there exists no standard approach how to define synergies or coordinative structures for speech articulation and a widely accepted standard higher-level control concept for speech articulation still needs to be developed.

As mentioned above, a very comprehensive and detailed neural model for speech production is the DIVA model (Guenther, 2006), but even this model does not include a detailed muscle-based biomechanical model. This may result from the fact that in case of human sound production we do not find a one-to-one relation of muscles and speech sounds. Even the production of a single speech sound (a single configuration of speech articulators) requires a synergistic cooperation of different groups of muscles (cf. Dang and Honda, 2004) while for example in the case of birdsongs, sounds are producible by one-to-one innervations of vocal muscles (Adam et al., 2021).

Already at the end of the last century leading researchers in speech production stated: “It has been hoped for decades that speech synthesis based on articulatory geometry and dynamics would result in a breakthrough in quality and naturalness of speech synthesis, but this has not happened. It is now possible to generate high quality synthetic speech, such as with the Klatt synthesizer, by modeling only the properties (spectral, etc.) of the output signal.” (Wilhelms-Tricarico and Perkell 1997, p. 222). During the following decades the situation has not changed much for articulatory-acoustic speech synthesis, while the quality of acoustic corpus-based speech synthesis increased dramatically towards nearly natural (Zen et al., 2009; Kahn and Chitode, 2016, and see research goals in Figure 2). Thus, the problem of high-quality speech synthesis is solved from the viewpoint of engineering but increasing the quality of articulatory-acoustic speech synthesis should be a side product if more knowledge is available concerning natural movement generation for speech articulators and if the remaining research questions in aeroacoustic signal generation are solved.

But nevertheless, one remaining goal of modeling speech production including speech articulation will be to reach high-quality speech synthesis. To reach this goal will be a step-by-step procedure which concerns all domains of the speech production process: 1) More anatomical details needs to be included in modeling, e.g., concerning the 3D-shape of vocal tract cavities and its changes during speaking (e.g., Vampola et al., 2015; Traser et al. 2017; Birkholz and Drechsel, 2021). 2) More knowledge is needed concerning the neurophysiological processes of controlling the movements of vocal tract organs during speech production at a higher control level (e.g., Guenther, 2006; Bohland et al., 2010; Guenther and Vladusich, 2012) as well as at the neuromuscular level at which neural activation causes articulator motion (e.g. Buchaillard et al., 2009). 3) More knowledge is needed especially concerning the phonation process in order to be able to produce individual and naturally sounding voice qualities (e.g., Vasudevan et al., 2017; Maurerlehner et al., 2021). 4) More acoustic and aeroacoustic knowledge is needed for improving the quality of acoustic signal generation and signal modification within the vocal tract (Zappi et al., 2016; Schoder et al., 2020).

Last but not least an attempt will be made here to answer the three core questions raised in the abstract concerning improvement of the generated acoustic signal quality and concerning the biological realism of the control concept and concerning the articulatory model. 1) Currently there exists no concrete concept for modeling the underlying neural organization controlling speech articulation. This concept should be neurobiologically grounded, should be activated by linguistic information like a phonological sound chain augmented by prosodic information, and should generate a motor description of the utterance under production. This kind of information is generated by gesture scores as defined by Ramanarayanan et al. (2013). But a neurobiological realization and implementation of gesture scores for example in a neural speech production model as well as the realization of a comprehensive approach for generating muscle activation patterns from gesture scores and its incorporation in a complete sensorimotor model of speech production has not yet been realized yet. This should be one of the next steps in developing control concepts for articulatory speech synthesis and for developing speech production models in general.

2) A lot of approaches are available for modeling speech articulators like tongue, lips, velum etc. and their movements. There are geometrical, statistical, or biomechanical approaches as discussed in this paper. In contrast to geometrical and statistic approaches, which are already usable as part of articulatory speech synthesizers (e.g., Birkholz model, see VocalTractLab, 2021; Maeda model as part of the DIVA speech production model, see VTCalcs, 2021) the next step or goal should be to establish comprehensive biomechanical and neurobiologically plausible models capable of generating speech movements for a complete set of vocal tract articulators and thus to establish biomechanical models capable for generating a temporal succession of complete vocal tract cavity shapes (vocal tract geometries) as a basis for calculating the acoustic speech signal (e.g., Vogt et al., 2005; ArtiSynth, 2021). 3) Beside modeling articulatory movement patterns and their control, the improvement of the acoustic signal generation within the vocal tract is a further goal. As already discussed above a lot of work is currently done in order to improve the modeling of the primary sound source by developing complex self-oscillating vocal fold models and their interaction with the aerodynamic system, in order to improve the modeling of secondary sound sources by unfolding the aeroacoustic principles of noise generation which in principle occur at all vocal tract constrictions, and in order to improve the quality of the acoustic signal modification in the vocal tract by using sophisticated acoustic/aeroacoustic modeling approaches.

But all these steps increase the computational costs and thus bring us far away from real-time applications which of course should be a further goal even for articulatory speech synthesis. Beside increasing naturalness of speech synthesis by using exclusively biologically motivated production principles the ultimate benchmark for the quality of articulatory speech synthesizers is the naturalness of the generated acoustic speech signal. While for example articulatory movement patterns like mouth or tongue movements may be assessed relatively easily as natural by our visual perceptual system this is not the case for the auditory perception of acoustic speech signals. Thus, the ultimate challenge for the developers of speech synthesis systems is the auditory evaluation of the produced speech signals and as each developer knows, the generation of not only understandable but also natural speech is the most complex and most challenging goal in developing and improving speech synthesis systems.
